# Regulation of KSHV Latency and Lytic Reactivation

**DOI:** 10.3390/v12091034

**Published:** 2020-09-17

**Authors:** Grant Broussard, Blossom Damania

**Affiliations:** 1 Lineberger Comprehensive Cancer Center, University of North Carolina at Chapel Hill, Chapel Hill, NC 27599, USA; gsbrou@email.unc.edu; 2 Curriculum in Genetics and Molecular Biology, University of North Carolina at Chapel Hill, Chapel Hill, NC 27599, USA; 3 Department of Microbiology and Immunology, University of North Carolina at Chapel Hill, Chapel Hill, NC 27599, USA

**Keywords:** Kaposi’s sarcoma-associated herpesvirus (KSHV), latency, lytic cycle, reactivation, viral life cycle

## Abstract

Kaposi’s sarcoma-associated herpesvirus (KSHV) is associated with three malignancies— Kaposi’s sarcoma (KS), primary effusion lymphoma (PEL), and multicentric Castleman’s disease (MCD). Central to the pathogenesis of these diseases is the KSHV viral life cycle, which is composed of a quiescent latent phase and a replicative lytic phase. While the establishment of latency enables persistent KSHV infection and evasion of the host immune system, lytic replication is essential for the dissemination of the virus between hosts and within the host itself. The transition between these phases, known as lytic reactivation, is controlled by a complex set of environmental, host, and viral factors. The effects of these various factors converge on the regulation of two KSHV proteins whose functions facilitate each phase of the viral life cycle—latency-associated nuclear antigen (LANA) and the master switch of KSHV reactivation, replication and transcription activator (RTA). This review presents the current understanding of how the transition between the phases of the KSHV life cycle is regulated, how the various phases contribute to KSHV pathogenesis, and how the viral life cycle can be exploited as a therapeutic target.

## 1. Introduction

Herpesviruses have been co-evolving with their animal hosts for millions of years. The life cycle of these viruses represents a compromise with the host immune system, forged by selective pressures to produce viral progeny while minimizing damage to the host that would compromise viral fitness. The success of this strategy is apparent, evidenced by the lifelong nature of herpesvirus infections. Periodic reactivation from a quiescent state into a replicative phase allows the production of large amounts of infectious virions, ensuring the survival of the virus within the host and the transmission of the virus to new hosts. Herpesvirus infections are treatable by inhibition of replication during the lytic phase but are ultimately incurable due to established latency. A greater understanding of the regulation of this life cycle could provide insights into how to therapeutically disrupt it, leading to new treatments for herpesvirus infections and the diseases they cause.

Kaposi’s sarcoma-associated herpesvirus (KSHV) is associated with three malignancies—Kaposi’s sarcoma (KS), primary effusion lymphoma (PEL), and multicentric Castleman’s disease (MCD). The pathogenesis of each of these diseases involves contributions from factors associated with both the lytic and latent phases of the viral life cycle [1]. The latency program of KSHV is a quiescent state involving the expression of relatively-few genes. During latency, KSHV persists by replication and segregation of extrachromosomal viral episomes to daughter cells by host cell machinery during mitosis. In response to external factors, latency can be disrupted by a bout of lytic reactivation, which triggers expression of the entire viral genome, replication of viral episomes via a rolling-circle mechanism, and production of infectious virions [2]. Since this phase of the viral life cycle results in the expression of many viral proteins, thereby exposing the virus to detection by the host immune system, entry into the lytic cycle is tightly controlled by a wide variety of mechanisms [3]. This review will summarize those mechanisms, illustrate how they contribute to KSHV pathogenesis, and address their potential as therapeutic targets.

## 2. Mechanisms Regulating the Establishment of KSHV Latency

### 2.1. Initial Establishment of the Latent Epigenetic State

**Figure 1:**
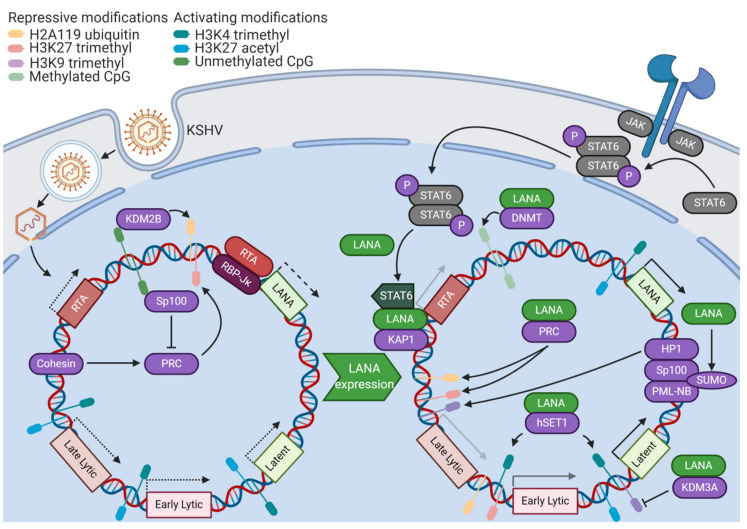
The Kaposi’s sarcoma-associated herpesvirus (KSHV) genome enters a latent state after de novo infection. In some cells, early expression of lytic genes such as replication and transcription activator (RTA) triggers expression of the master organizer of latency, latency-associated nuclear antigen (LANA). LANA recruits many components of the host epigenetic machinery to promote the formation of latent KSHV episomes. A pattern of transcriptionally-permissive histone modifications across the KSHV genome gives way to a generally-repressive chromatin state, sparing robust latent gene expression. Lytic gene expression becomes minimal but poised for upregulation upon reactivation.

KSHV latency relies upon a reliable, but reversible, control of gene expression. Thus, it is unsurprising that epigenetic modifications of the viral genome and its associated histones play a critical role (Figure 1). However, the KSHV genome enters the host cell nucleus after de novo infection in an epigenetically-naïve state [4]. Cellular epigenetic machinery is essential for the conversion of this transcriptionally-permissive genomic landscape into a fully chromatinized, latent KSHV episome. Due to the initially-open chromatin landscape, some lytic proteins, such as the master lytic switch replication and transcription activator (RTA), are first expressed at a low level. This early expression contributes to an initial wave of activating H3K4 trimethylation and H3K27 acetylation marks across the KSHV genome [5]. A binding site for a transactivation cofactor of RTA, recombination signal binding protein for immunoglobulin κ J region (RBP-Jκ), is found in the promoter region of the master organizer of KSHV latency, latency-associated nuclear antigen (LANA). Thus, paradoxically, early lytic gene activity promotes the transcription of factors essential for the establishment of KSHV latency [6]. However, it is important to note that latency can be established independently of RTA activity, as a KSHV RTA deletion mutant can establish latency with robust LANA expression. This is likely mediated by the transactivation of the LANA promoter by host transcription factors [7]. After 24 to 72 h, the epigenetic profile of the KSHV genome shifts from activating marks towards repressive H3K27 trimethylation and H2AK119 ubiquitylation marks. These repressive histone modifications are associated with the activity of Polycomb repressive complexes 1 and 2 (PRC1/2) [5]. Unlike closely-related herpesviruses, KSHV has a very GC-rich genome. Unmethylated CpG islands in the KSHV genome serve as binding sites for lysine demethylase 2B (KDM2B), a PRC1 component, contributing to the deposition of silencing H2AK119 ubiquitin modifications [8]. Cohesin, a protein complex involved in the formation of DNA loops, has been shown to be essential for the proper recruitment of PRC components, establishment of repressive histone marks, and eventual silencing of RTA expression on epigenetically-naïve KSHV genomes [9]. The establishment of these epigenetic features sets up the parameters for the KSHV latency program.

### 2.2. LANA

LANA plays a wide variety of crucial roles in the initial establishment of KSHV latency (Figure 1). Many of the functions of LANA are dependent on its ability to recruit host machinery to the viral genome. LANA has been shown to interact with DNA methyltransferases and may recruit DNA methyltransferase 3A (DNMT3A) to broadly silence gene activity on the initially-unmethylated KSHV genome [10]. LANA recruits PRC components to lytic promoters following de novo infection, functioning as a transcriptional repressor of lytic genes [11]. LANA is also able to recruit Krüppel-associated box-associated protein 1 (KAP1), a transcriptional repressor, to the RTA promoter, shutting down the initial wave of lytic activity [12]. KAP1 then goes on to associate with most KSHV lytic gene promoters, triggering chromatin condensation and silencing gene expression [13]. LANA stimulates protease-mediated cleavage of nuclear signal transducer and activator of transcription 6 (STAT6). This cleavage removes the transactivation domain of STAT6, causing it to act instead as a transcriptional repressor of the RTA promoter [14]. In addition to antagonizing lytic gene expression, LANA also promotes the expression of latent genes. By recruiting human suppressor of variegation, enhancer-of-zeste, and trithorax domain containing protein 1 (hSET1) to viral promoters, LANA contributes to the methylation of H3K4, ensuring that all latent and some early lytic genes are poised for active transcription [15]. LANA contributes to the removal of heterochromatic H3K9 marks from latent and lytic immediate early promoters through the recruitment of lysine demethylase 3A (KDM3A) [16]. Through interactions with the host epigenetic machinery, LANA establishes a core feature of KSHV latency, which is a transcriptional state of robust latent gene expression where the genome is poised for lytic gene expression in the event of reactivating stimuli.

In addition to directly interacting with viral promoters, LANA contributes to KSHV latency establishment through posttranslational modification of host chromatin assembly factors. Speckled protein 100 (Sp100) is a component of the promyelocytic leukemia nuclear body (PML-NB) complex, which has been shown to have roles in repressing viral gene expression through association with heterochromatin protein 1 (HP1) [17]. LANA promotes the addition of a 10 kilodalton small ubiquitin-like modifier (SUMO) protein tag to Sp100 in a process known as SUMOylation. SUMOylation has been shown to alter protein localization, activity, or stability through alteration of protein–protein interaction surfaces [18]. In response to LANA-mediated SUMOylation, Sp100 is relocalized to the insoluble chromatin fraction (where PML–NB complexes reside). The depletion of Sp100 has been shown to accelerate the deposition of repressive H3K27 trimethylation marks [19]. Since Speckled family proteins have been shown to exhibit broad binding activity to unmethylated CpG islands, it is possible that soluble Sp100 may compete with PRC2 components for binding to these sites, antagonizing chromatin condensation [20]. The dramatic re-localization of soluble Sp100 by LANA occurs in the same temporal window as the broad H3K27 trimethylation of the KSHV genome, suggesting a role in the establishment of latency [19]. In addition to contributing to SUMOylation of other proteins, LANA is SUMOylated itself, enabling several of its protein–protein interactions with cellular complexes mediating mitosis, DNA unwinding, and mRNA processing [21]. LANA is deSUMOylated by SUMO/sentrin-specific peptidase 6 (SENP6), a process which decreases the stability of LANA and interferes with the establishment of latency. Accordingly, LANA directly binds the SENP6 promoter and reduces its expression, ensuring that LANA accumulates and contributes to the establishment of latency without interference from host deSUMOylation machinery [22]. Another posttranslational modification of LANA that stimulates the establishment of latency is arginine methylation. Protein arginine methyltransferase 1 (PRMT1) augments LANA histone binding, and a methylation mimetic mutant of LANA induces tighter control of lytic gene expression [23]. The many layers of regulation surrounding LANA highlight its critical role as a master organizer of latency.

## 3. Mechanisms Regulating the Maintenance of KSHV Latency

### 3.1. LANA

**Figure 2:**
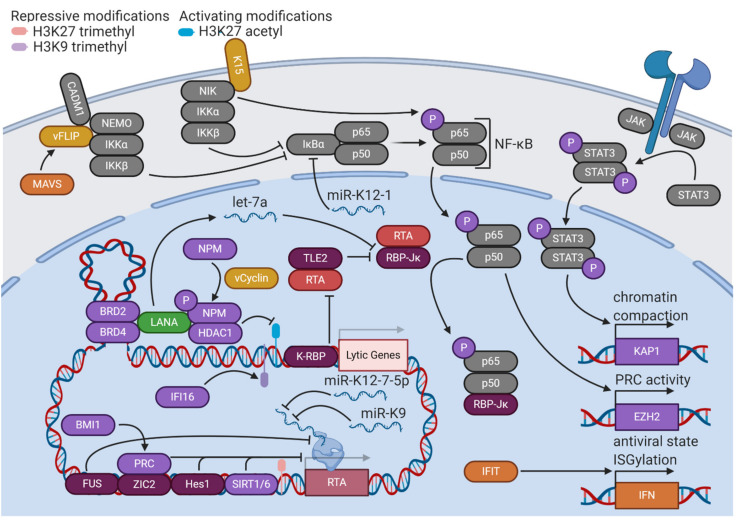
KSHV modulates host signaling pathways, chromatin structure, and gene expression to maintain latency. Viral proteins and miRNAs stimulate latency-promoting signaling pathways and chromatin modifiers. The expression and function of RTA is restricted by a variety of proteins and miRNAs from the virus and the host. LANA promotes repressive histone modifications and regulatory loops. Lytic gene expression is minimal but poised for reactivation upon shifts in the relative activities of RTA and LANA.

Once latency has been established, LANA plays essential roles in maintaining the latency state in cooperation with other KSHV latent gene products (Figure 2). LANA and viral Fas-associated protein with death domain (FADD)-like interleukin-1β-converting enzyme (FLICE)-like inhibitory protein (vFLIP) stimulate the upregulation of enhancer of zeste homolog 2 (EZH2), the H3K27 methyltransferase of the PRC2 complex, by upregulating the nuclear factor κ-light-chain-enhancer of activated B cells (NF-κB) pathway [24]. This upregulation further solidifies a repressive chromatin state with the expression of only a few viral genes. Viral cyclin (vCyclin), a homolog of cellular cyclin D2, phosphorylates threonine 199 of nucleophosmin (NPM), a histone chaperone that plays roles in transcriptional control and chromatin organization. This phosphorylation enables LANA binding, an interaction that is implicated in the promotion of latency, as the depletion or dephosphorylation of NPM is associated with KSHV reactivation [25]. LANA also complexes with DNA looping factors to maintain latency, such as bromodomain-containing proteins 2 and 4 (BRD2/4). Pharmacological disruption of BRD2 and BRD4 leads to the loss of a DNA loop tethering the latent and lytic control regions of the KSHV genome, triggering reactivation [26]. The critical role of chromatin organization in the maintenance of KSHV latency is evident, as other host factors, such as B cell-specific Moloney murine leukemia virus integration site 1 (BMI1), a PRC1 complex member, and tousled-like kinase 2 (TLK2) are implicated in restriction of KSHV reactivation [27,28].

### 3.2. NF-κB Signaling

KSHV latency is heavily dependent upon the activation of specific cellular signaling pathways (Figure 2). The NF-κB pathway is constitutively active in KSHV-infected cells and is implicated in the control of latency [29]. The NF-κB transcription factor is composed of the p65 and p50 subunits, both of which are sequestered in an inactive form in the cytoplasm bound to nuclear factor of κ light polypeptide gene enhancer in B-cells inhibitor α (IκBα). In response to an activating stimulus, the IκB kinase (IKK) complex (composed of regulatory subunit NF-κB, essential modulator (NEMO), and catalytic subunits IKKα and IKKβ) phosphorylates IκBα, leading to its ubiquitination and degradation. The NF-κB transcription factor is then free to translocate to the nucleus and transactivate many downstream target genes [30].

Activated NF-κB forms a complex with RTA cofactor RBP-Jκ, thus competitively restricting KSHV lytic activity [31]. KSHV infection activates IKKβ and p65 serine 536 phosphorylation, which together increase NF-κB target binding affinity and provide a constitutive stimulatory signal for the pathway [32]. This effect is mediated primarily through two KSHV latent gene products—K15 and vFLIP. K15, a transmembrane protein expressed at low levels during latency, contributes to NF-κB activation by recruiting a complex composed of NF-κB-inducing kinase (NIK) (another NF-κB stimulatory factor), IKKα, and IKKβ, which results in the serine 536 phosphorylation of p65 [33,34]. vFLIP forms a complex with cell adhesion molecule 1 (CADM1) and NEMO at the plasma membrane to promote IKK activation and subsequent NF-κB-mediated restriction of lytic reactivation [35]. Additionally, K15 and vFLIP stimulate mucosa-associated lymphoid tissue lymphoma translocation protein 1 (MALT1) protease activity, a primary driver of NF-κB activation in lymphocytes. K15- and vFLIP-stimulated NF-κB activation was shown to be dependent on MALT1, and inhibition of MALT1 protease activity stimulated KSHV reactivation in PEL cell lines [36]. A KSHV micro-RNA (miRNA), miR-K1, has also been demonstrated to upregulate NF-κB by targeting the 3′ untranslated region (UTR) of IκBα mRNA, leading to lower levels of inhibitor and release of NF-κB [37]. The many interactions between the NF-κB pathway and viral factors demonstrate its essentiality to the KSHV life cycle.

### 3.3. STAT3

Another cellular signaling pathway that has been implicated in KSHV latency is STAT3 (Figure 2). When STAT3 is phosphorylated by Janus kinases (JAKs) associated with a receptor tyrosine kinase, it dimerizes and translocates to the nucleus to stimulate transcription of target genes. STAT3 is constitutively active in cells latently infected with KSHV. If this constitutive activation is interrupted by knockdown of STAT3, levels of transcriptional repressor KAP1 decrease, and lytic reactivation ensues [38]. Chemical inhibition of STAT3 tyrosine 705 phosphorylation also induces lower levels of KAP1 and higher levels of KSHV lytic activity [39].

### 3.4. Immune Sensors

Cellular immune defenses seek to limit viral gene expression and replication that would be detrimental to the survival of the host cell. KSHV exploits these host pathways to maintain latency (Figure 2). Interferon-γ-inducible protein 16 (IFI16), an innate immune DNA sensor, complexes with lysine methyltransferases suppressor of variegation 3-9 homolog 1 (SUV39H1) and G9a-like protein (GLP) to promote H3K9 trimethylation of the KSHV genome. These repressive marks subsequently recruit HP1α to mediate heterochromatinization and silencing of viral gene expression [40]. The expression level of vFLIP protein, a crucial mediator of KSHV latency, is regulated by a different cellular immune defense protein, mitochondrial antiviral signaling (MAVS) adaptor. vFLIP is targeted to the MAVS signaling complex on peroxisomes, where it is stabilized through protein–protein interaction [41]. The inflammasome is another immune defense pathway that controls KSHV reactivation. Inflammasomes are signaling complexes that oligomerize in response to a foreign antigen (such as viral DNA) and recruit caspases to initiate the production of inflammatory cytokines and/or cell death [42]. Nucleotide-binding oligomerization domain (NOD)-like receptor (NLR) family pyrin domain containing protein 1 (NLRP1) is an inflammasome component that has been demonstrated to restrict KSHV reactivation from latency, as well as prevent mature virion production [43]. Thus, host cellular immune defenses facilitate KSHV’s unique life cycle.

### 3.5. Interferon

Other host factors promote latency through the activity of interferons (IFNs), signaling proteins produced in response to infection that promote antiviral activities such as reduction of protein synthesis and apoptosis (Figure 2) [44]. The interferon-induced protein with tetratricopeptide repeats (IFIT) family of proteins are induced by KSHV infection in epithelial cells. These proteins upregulate IFNβ and 2′,5′-oligoadenylate synthetase (OAS) proteins to promote the degradation of viral RNA. Loss of IFIT1, IFIT2, and IFIT3 leads to broad increases in lytic gene expression and subsequent virion production, implicating them in control of KSHV latency [45]. Interferon stimulation also changes cellular patterns of posttranslational modifications, particularly through the induction of interferon-stimulated gene 15 (ISG15) conjugation. “ISGylation” appends a 17 kilodalton protein tag to proteins synthesized under conditions of interferon induction, potentially shuttling dangerous proteins towards lysosomal degradation during viral infection [46]. Knockdown of ISG15 in cells latently infected with KSHV results in increased levels of lytic reactivation, implicating this antiviral defense mechanism in the maintenance of latency [47]. ISG15 and another interferon-stimulated gene, ISG20, have been shown to maintain latency by promoting expression of miR-K1, a viral miRNA which regulates the NF-κB pathway [48]. IFNγ broadly inhibits KSHV gene expression and restricts the production of progeny virions [49]. A role for IFNγ in control of KSHV latency is reasonable, as a close KSHV relative, murine gammaherpesvirus 68 (MHV68), reactivates from latency more readily in mice deficient in IFNγ or its receptor [50]. In addition to cell-intrinsic defenses, the systemic immune system also contributes to the stabilization of KSHV latency. CD4+ T cells engage in contact-dependent, MHC-independent inhibition of lytic replication in infected B cells, demonstrating the key role of systemic responses in shaping the course and outcome of KSHV infection [51].

### 3.6. Suppression of RTA

KSHV latency is disrupted by the activity of the lytic master switch protein RTA. Accordingly, the latency program involves many layers of restriction on the expression of RTA, implemented by a variety of host and viral factors (Figure 2). In addition to regulating broad repression of KSHV lytic gene expression, host epigenetic factors target the RTA promoter to prevent the initiation of the lytic cascade. The depletion of EZH2, the methyltransferase of the PRC2 complex, leads to RTA expression and lytic reactivation [52]. Zinc finger protein of the cerebellum 2 (ZIC2) tethers PRC2 to the RTA promoter (as well as other early lytic promoters), ensuring repressive H3K27 trimethylation is maintained. However, RTA can trigger the degradation of ZIC2 by acting as an E3 ubiquitin ligase, suggesting a mutual regulatory mechanism [53]. Sirtuin 1 (SIRT1) is a class III histone deacetylase (HDAC) that binds to the RTA promoter, contributing to increased repressive H3K27 trimethylation, decreased activating H3K4 methylation, and overall decreased promoter activity. Additionally, SIRT1 can physically interact with RTA, inhibiting the transactivation of its own promoter and other lytic promoters [54]. Sirtuin 6 (SIRT6) is also capable of binding the RTA promoter, inhibiting lytic reactivation and viral gene expression [55]. Epigenetic control of RTA expression is essential for restriction of KSHV reactivation.

Host factors not associated with epigenetic processes exert control over RTA activity through different mechanisms. Fused in sarcoma (FUS), a DNA/RNA binding protein, inhibits expression from the RTA promoter by preventing serine-2 phosphorylation of the C-terminal domain of RNA polymerase II [56]. Surprisingly, KSHV RTA-binding protein (K-RBP) does interfere with RTA’s function, but not through direct physical interaction. K-RBP binds to viral promoters through its zinc finger domain, preventing transactivation by RTA and restricting lytic gene expression [57]. Transducin-like enhancer protein 2 (TLE2) competes with the principal coactivator of RTA, RBP-Jκ, for access to the proline-rich domain of RTA, ultimately interfering with its transactivation ability [58]. Hairy and enhancer of split protein 1 (HES1), a downstream effector of Notch signaling, binds to and transcriptionally represses the RTA promoter. Lytic reactivation upregulates Jagged-1 (JAG1), a Notch ligand, such that the neighbors of reactivating cells experience upregulation in Notch signaling. Thus, it is plausible that this feedback mechanism limits the number of cells undergoing lytic reactivation in a certain population, ensuring that latency is the predominant cell state [59].

KSHV also controls the expression of RTA by modulating expression of cellular miRNAs. LANA expression stimulates the expression of let-7a, a host miRNA that targets the 3′ UTR of RBP-Jκ. The resulting decreased expression of RBP-Jκ, combined with LANA competitively binding the remaining RBP-Jκ, interferes with the transactivation capabilities of RTA [60]. In addition to modulating cellular miRNAs, KSHV encodes its own set of viral miRNAs. These miRNAs have broadly inhibitory effects on gene transcription. The deletion of the KSHV miRNA locus leads to a more open chromatin landscape with decreased H3K9 methylation, increased H3 acetylation, and a loss of DNA methylation. miR-K12-4-5p specifically targets retinoblastoma-like protein 2 (RBL2), a repressor of DNMT3A transcription [61]. In addition to globally-repressive effects on viral gene expression, KSHV miRNAs also have functions that are targeted to RTA and restriction of lytic activation. Two KSHV miRNAs, miR-K12-7-5p and miR-K9, have been reported to target the 3′ UTR of RTA mRNA, causing degradation in a seed sequence-specific manner [62,63]. Additionally, KSHV miR-K3 regulates levels of RTA expression by targeting the 3′ UTR of nuclear factor I/B (NFIB), a transcriptional activator of the RTA promoter [64]. The fact that KSHV encodes viral factors which specifically control the activity of RTA highlights its essentiality as the trigger of lytic reactivation.

## 4. Mechanisms Regulating KSHV Reactivation from Latency

### 4.1. RTA

**Figure 3:**
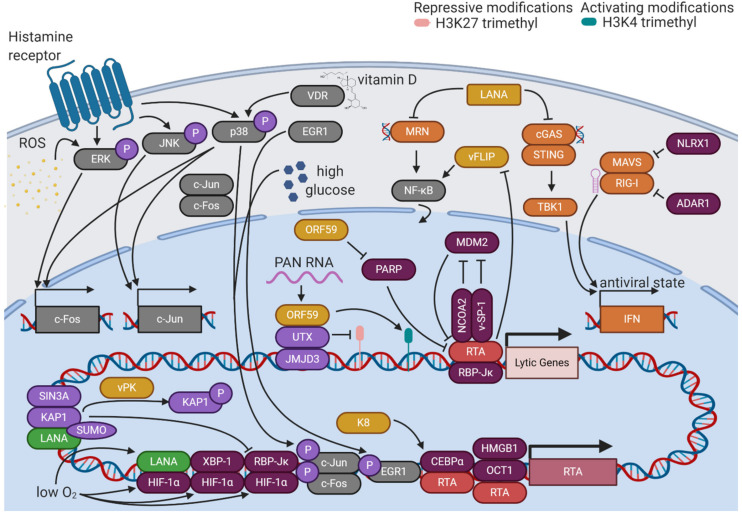
Environmental stimuli alter regulation of RTA to promote KSHV reactivation. Host signaling pathways integrate information about the cellular microenvironment that affects the expression and activity of RTA. Lytic viral proteins promote KSHV reactivation in a feed-forward loop. Various isoforms and posttranslational modifications of LANA switch it from an organizer of latency to a facilitator of the lytic cycle. The cell-intrinsic immune system is antagonized by host and viral factors to augment viral reactivation.

KSHV reactivation from latency is initiated when various stimuli promote the expression of RTA, the master lytic switch protein (Figure 3). Once RTA accumulates to sufficient levels, it can outcompete LANA for binding to its major cofactor, RBP-Jκ, allowing RTA to target 100 distinct sites in the KSHV genome and transactivate 34 lytic genes [65]. One of the most critical promoters that RTA targets is its own, which it does in association with a complex of octamer protein 1 (Oct-1) and high mobility group box 1 protein (HMGB1) or in association with CCAAT/enhancer-binding protein α (CEBPα) [66,67]. RTA transactivation of the RTA promoter creates a feed-forward loop that overcomes the repressive latent state and ensures a continual increase in lytic gene expression. Certain lytic gene products cooperate with RTA to maintain this feed-forward loop. K8 stabilizes CEBPα, promoting RTA transactivation of its own and other lytic promoters [67]. KSHV open reading frame 57 (ORF57), another lytic protein, binds directly to RTA and stimulates transcription from its promoter [68]. Thus, KSHV reactivation sustains itself through the contributions of lytic proteins to the regulation of the lytic cycle.

In addition to its roles as a transcription factor, RTA promotes KSHV lytic activation through its role as an E3 ubiquitin ligase. E3 ubiquitin ligases catalyze the addition of ubiquitin protein tags to their substrates, marking them for proteasomal degradation. One ubiquitination substrate of RTA is K-RBP, a DNA-binding protein that restricts the binding of RTA to lytic promoters [69]. RTA targets latent KSHV viral proteins as well, promoting proteasomal degradation of vFLIP to antagonize latency-promoting signaling pathways [70]. The abundance of RTA itself is subject to regulation by the ubiquitin proteasome system. Nuclear receptor coactivator 2 (NCOA2) stabilizes RTA by binding its protein abundance regulatory signal (PARS) domain [71]. This domain is typically targeted by the cellular E3 ubiquitin ligase mouse double minute 2 homolog (MDM2), but competition by NCOA2 ensures the accumulation of RTA and activation of the lytic cycle [72]. Viral factors also posttranslationally modulate the levels of RTA activity. A small peptide encoded by KSHV, viral small peptide 1 (vSP-1), has been shown to bind the PARS domain, preventing RTA degradation and promoting the lytic cycle [73]. KSHV ORF59 degrades poly(ADP-ribose) polymerase 1 (PARP-1), a posttranslational modifier that interferes with RTA activity [74]. Multiple levels of regulation of the activity and abundance of RTA allows for precise control of the reactivation of the virus.

RTA activation responds to various environmental stimuli. One of the most prominent of these is hypoxia. KSHV lytic activity was first suspected to be linked to hypoxia based on the clinical features of KS. KS lesions, whose formation and progression are linked to the activities of KSHV lytic proteins, are often found on the distal portions of the body where oxygen levels are low. Hypoxia induces the activity of hypoxia-inducible factors (HIFs) by stabilizing HIF-1α, the regulatory unit of the HIF complex. Under normoxic conditions, HIF-1α is degraded due to hydroxylation of its oxygen-dependent degradation (ODD) domain. When oxygen levels are low, HIF-1α is not degraded, and can interact with cellular factors like X-box binding protein 1 (XBP-1) to transactivate the RTA promoter and spur KSHV lytic activity [75]. The active spliced form of XBP1, XBP1-s, is upregulated during B cell differentiation, implementing another cellular environmental stimulus in direct control of RTA expression [76]. RTA activity is also sensitive to calcium signaling, as ionomycin, a calcium ionophore, potently induces RTA expression and lytic reactivation [77]. Thus, KSHV senses and responds to its environment through the relative activities of viral and host factors.

### 4.2. Suppression of LANA

Since LANA typically functions antagonistically to KSHV reactivation, certain cellular contexts and posttranslational modifications alter its activity to promote reactivation (Figure 3). While phosphorylation of serine 10 and threonine 14 residues by host kinases such as casein kinase 1 (CK1), glycogen synthase 3 (GSK3), or ribosomal S6 kinase 3 (RSK3) promotes the interaction of LANA with chromatin, serine 205/206 phosphorylation by pro-viral integration site for Moloney murine leukemia virus-1 or 3 (PIM1/3) counteracts LANA-mediated repression of lytic gene transcription [78,79]. Lysine acetylation of LANA increases after lytic induction with sodium butyrate, an HDAC inhibitor, suggesting a potential regulatory role of this modification [80]. Posttranslational modifications of LANA also play a major role in hypoxia-mediated KSHV reactivation. LANA can function as a transcriptional activator under these conditions, binding HIF-1α and transactivating the RTA promoter [81]. DeSUMOylation of both LANA and KAP1 during hypoxia leads to the dissociation of the repressive LANA/KAP1/SIN3A complex [82]. The release of repressive KAP1 enables the binding of an HIF-1α/RBP-Jκ complex to the RTA promoter, augmenting its expression [83]. In contrast to the function of nuclear LANA, cytoplasmic isoforms of LANA mediate lytic reactivation by antagonizing cellular DNA sensors. Cytoplasmic LANA directly binds cyclic GMP-AMP synthase (cGAS), interfering with downstream cGAS-stimulator of interferon genes (STING) interferon induction [84]. Cytoplasmic LANA can also bind the Mre11-Rad50-NBS1 (MRN) complex, preventing its recognition of cytoplasmic DNA and subsequent activation of the NF-κB pathway [85]. Through various isoforms and modifications, LANA switches roles from the master organizer of latency to a facilitator of the lytic cycle.

### 4.3. MAPK Signaling Pathways

Environmental control of KSHV reactivation is mediated through the effects of stimuli on cellular signaling pathways (Figure 3). The major cellular signaling pathways that control KSHV reactivation are mitogen-activated protein kinase (MAPK) signaling cascades. Inhibition of the extracellular signal-regulated kinase (ERK) pathway, the Jun N-terminal kinase (JNK) pathway, or the p38 pathway leads to defects in KSHV reactivation [86]. These three pathways converge on regulation of the activity of a transcription factor called activator protein 1 (AP-1). AP-1 is a heterodimer of two proteins—c-Fos and c-Jun. ERK activation can stimulate c-Fos transcription through phosphorylation of transcription factors known as ternary complex factors (TCFs). p38 activation can also lead to TCF phosphorylation and c-Fos transcription, while additionally phosphorylating activating transcription factor 2 (ATF2) to promote c-Jun transcription. Activated JNK can directly phosphorylate c-Jun, promoting transcriptional activation upon heterodimerization with c-Fos and stimulation of the c-Jun promoter [87]. AP-1 activity is required for the activation of the RTA promoter [86]. Kirsten rat sarcoma viral oncogene homolog (KRAS) upregulates RTA expression through the rapidly-accelerated fibrosarcoma (RAF)/mitogen-activated protein kinase (MEK)/ERK pathway [88]. Early growth response protein 1 (EGR1), a phosphorylation target of the p38 pathway, binds to and promotes transcriptional activity at the RTA promoter [89]. Through regulation of RTA, the host MAPK signaling pathways function as key triggers of the KSHV lytic cycle.

MAPK pathways play essential roles in the cellular response to reactive oxygen species (ROS). Free radical oxygen species such as superoxide, hydroxyl radicals, and singlet oxygen, as well as partially-reduced oxygen in the form of hydrogen peroxide, have the potential to be highly damaging to cellular components through oxidation of biomolecules [90]. ROS also serves as a trigger for KSHV reactivation, indicating that the virus responds to host cell stress by sensing stress signaling pathways. ROS induces the expression of RTA, as well as downstream lytic genes ORF57, ORF59, K8, and ORF65, in a dose-dependent manner [91]. ROS stimulates phosphorylation of ERK, triggering KSHV lytic reactivation [92]. ROS are also implicated in the mechanism of chemical induction of KSHV lytic reactivation by tetradecanoyl phorbol acetate (TPA). TPA is known to be dependent on the activity of protein kinase C δ (PKCδ), which has also been demonstrated to increase cellular ROS levels [92]. Forkhead box protein O1 (FOXO1), a significant stimulator of cellular redox defense, restricts KSHV lytic reactivation, providing further evidence of the key role of ROS in the KSHV life cycle [93].

KSHV also responds to other environmental stimuli through the activities of cellular signaling pathways. Histamine signaling has been shown to promote KSHV lytic reactivation through a mechanism involving the MAPK and phosphatidylinositol 3-kinase (PI3K)/protein kinase B (PKB) signaling cascades. HIV-positive patients with KSHV co-infection have significantly increased levels of histamine in plasma and saliva compared to those without KSHV infection, indicating that this signaling molecule may be relevant to the systemic manifestations of viral infection [94]. PEL cells treated with 1,25-dihydroxyvitamin D3, the biologically-active form of vitamin D, exhibited increased levels of KSHV reactivation in a vitamin D receptor (VDR) and p38-dependent manner, implicating this signaling axis in the regulation of the KSHV lytic cycle [95]. High glucose has also been shown to stimulate KSHV reactivation through AP-1 activation, which may contribute to the increased severity of KSHV-associated disease in diabetic patients [96]. The broad variety of signals integrated by MAPK signaling pathways explains the diversity of environmental triggers of KSHV reactivation.

### 4.4. Epigenetic Mediators

KSHV encodes various lytic factors that modify the epigenetic state of the viral genome as the lytic cycle progresses to promote viral gene expression (Figure 3). ORF36, also known as viral protein kinase (vPK), phosphorylates KAP1, releasing it from chromatin and subverting its repressive function [13]. ORF59 binds protein arginine methyltransferase 5 (PRMT5), sequestering it from its binding partner coordinator of PRMT5 and differentiation stimulator (COPR5). The disruption of this interaction decreases repressive H4R3 methylation and increases activating H3K4 trimethylation, promoting a more open chromatin landscape to augment viral gene expression [97]. Polyadenylated nuclear (PAN) RNA, a highly-expressed non-coding KSHV lytic RNA, facilitates interaction between ORF59, histone demethylases ubiquitously transcribed tetratricopeptide repeat X chromosome (UTX) and Jumonji-domain-containing 3 (JMJD3), and the viral genome. The subsequent reduction in H3K27 trimethylation, particularly at the ORF50 promoter, promotes robust lytic gene expression [98,99]. Conversely, KSHV lytic protein K8 acts as a modulator of KSHV lytic reactivation by recruiting HDACs to the RTA promoter to repress transcriptional activity, potentially serving as a brake on runaway lytic activity [100]. As reactivation progresses, lytic proteins shape the epigenetic state of the KSHV genome to promote completion of the viral life cycle.

### 4.5. Environmental Stimuli

The cellular microenvironment can shape the KSHV epigenome in ways that facilitate reactivation from latency. ER stress can induce cleavage of Rad21, a member of the cohesin complex, leading to dramatic changes in the structure of the KSHV genome. The resulting loss of potentially-inhibitory DNA loops triggers KSHV reactivation [101]. Cellular differentiation, which also involves dramatic epigenetic reorganization, was shown to be a trigger for KSHV reactivation in a keratinocyte raft model of KSHV infection [102]. *Pseudomonas aeruginosa*, an opportunistic pathogen commonly found in immunocompromised patients, produces quorum-sensing molecules for intercellular communication, which have been shown to induce KSHV lytic gene expression [103]. *Mycobacterium tuberculosis* co-infection has been linked to pulmonary KSHV infections. ESAT-6, a tuberculosis virulence factor, has been shown to induce viral reactivation in KSHV-infected pulmonary endothelial cells [104]. Periodontal pathogens in the oral microenvironment secrete short-chain fatty acids that inhibit class I and II HDACs and downregulate epigenetic modulators like SIRT1, EZH2, and SUV39H1, creating an open chromatin landscape and a higher propensity for KSHV lytic reactivation [105]. Other metabolic end products of common periodontal pathogens have been demonstrated to induce reactivation of KSHV via stimulation of the p38 MAPK pathway [106]. *Staphylococcus aureus* commonly infects the oral cavity of HIV-positive individuals, and its pathogen-associated molecular patterns (PAMPs) have been found to stimulate lytic reactivation in KSHV-infected oral cells through the Toll-like receptor (TLR)-ROS and cyclin D1-Dicer-viral miRNA axis. The modulation of lytic activity in the oral body compartment is central to the productive transmission of KSHV [107].

### 4.6. Immune Regulators

Since the cellular intrinsic immune system broadly seeks to limit viral replication, many KSHV viral factors specifically antagonize viral sensing and antiviral defenses (Figure 3). Viral interferon regulatory factor 1 (vIRF1) blocks STING from binding TANK-binding kinase 1 (TBK1), interfering with cGAS-STING-mediated IFNβ induction and permitting higher levels of KSHV reactivation [108]. vIRF1 also binds HECT and RLD domain-containing E3 ubiquitin-protein ligase 5 (HERC5), an ISG15 E3 ligase, under conditions of interferon stimulation, decreasing total ISG15 conjugation and enabling robust lytic gene expression [47]. Late lytic gene products target IFI16, which typically senses pathogenic DNA and stimulates epigenetic silencing, for degradation, allowing lytic cycle activity to continue with an open chromatin landscape [40]. ORF63 inhibits the formation of inflammasome complexes by NLRP1, subverting its typical antiviral role and promoting KSHV reactivation [43]. By targeting the cellular immune system, KSHV creates a less hostile microenvironment for lytic reactivation.

Some immune pathways escape viral subversion and prevent runaway viral reactivation. MAVS and retinoic acid-inducible gene I (RIG-I) induce IFNβ through a mechanism involving RIG-I-dependent RNA sensing of specific KSHV mRNAs, tempering the progression of the lytic cycle [109,110]. However, certain host immune pathways play a pro-viral role in KSHV reactivation. TLRs respond to a variety of PAMPs by promoting inflammation and interferon production. TLR 7 and 8, which sense single-stranded RNA, have been shown to trigger reactivation of KSHV when stimulated by co-infection with vesicular stomatitis virus [111]. Thus, viral co-infection is yet another environmental stimulus that modulates the KSHV life cycle through effects on host signaling pathways. NLR family member X1 (NLRX1) is another host factor that facilitates KSHV reactivation from latency by downregulating IFNβ responses to viral activity [112]. Similarly, adenosine deaminase acting on RNA 1 (ADAR1) promotes KSHV reactivation from latency by blocking RIG-I-like receptors (RLRs) from sensing RNA duplexes, decreasing the interferon response [113]. KSHV depends on host factors to control the cellular immune response to its life cycle.

## 5. Contributions of the KSHV Latent and Lytic Cycles to KSHV-Associated Disease Pathology

**Figure 4:**
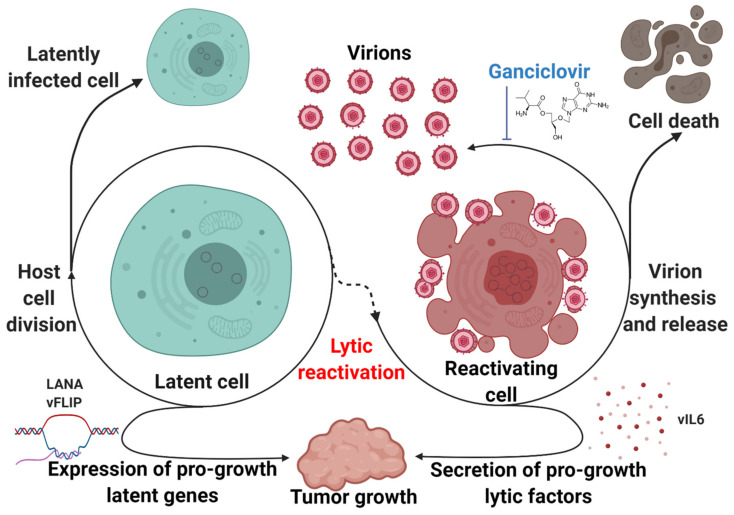
The latent and lytic stages of the KSHV life cycle drive pathogenesis. Latency maintains a population of infected cells by segregating KSHV episomes to daughter cells after mitosis. Lytic reactivation produces infectious virions that expand the pool of infected cells. Both latent and lytic factors promote tumor growth through viral oncogenes or paracrine factors. Lytic replication is sensitive to chemical inhibition, while latent episomes are difficult to target. Therapeutic interventions can force the virus out of latency, promoting cell death.

KSHV expresses genes in latency that have tumorigenic potential (Figure 4). B-cell specific expression of LANA leads to lymphoma, follicular hyperplasia, and increased germinal center formation in transgenic mice [114]. Similarly, vFLIP transgenic mice tend to develop lymphomas and other B-cell-driven tumors [115]. However, it has long been recognized that latency alone is not sufficient to explain the development of KSHV-associated disease. Complete lytic replication leads to the destruction of the host cell, but abortive lytic replication allows for the production of a subset of lytic viral genes without triggering cell death. These lytic viral genes encode for proteins that can enhance tumorigenesis through a paracrine mechanism. Lytic replication has been shown to play a key role in the biology of KSHV-associated malignancies. This concept was first proposed to explain the observation that treatment with ganciclovir, a viral DNA synthesis inhibitor, reduced the incidence of KS in a cohort of susceptible patients by 75% when administered orally and 93% when administered intravenously [116]. The introduction of the KSHV genome into certain cell types has been demonstrated to be sufficient for oncogenesis. Rat mesenchymal cells infected with KSHV undergo cellular transformation and efficiently induce KS-like tumors in nude mice. The resulting tumor cells are predominantly latently infected with an occasional heterogeneous expression of lytic genes, which is representative of the pathobiology of both KS and PEL [117]. Introduction of the KSHV genome into murine bone-marrow-derived cells, either by infection or transfection of a KSHV bacterial artificial chromosome, have also demonstrated efficient transformation and the establishment of KS-like tumors with the propensity to produce mature KSHV virions [118,119].

Known as spindle cells due to their characteristic morphology, KS tumor cells express a mixture of cell-type markers from endothelial, monocytic, and smooth muscle lineages [120]. Endothelial cells have thus been suspected as a spindle cell precursor. Lymphatic endothelial cells infected with KSHV can overcome replicative senescence in a vCyclin-dependent manner [121]. However, most KSHV-infected endothelial cell lineages demonstrate no growth advantage, tend to lose the viral genome, and demonstrate no transformation, suggesting that certain aspects of the in vivo microenvironment that drives the oncogenic potential of KSHV infection may not be accurately replicated in cell culture [122]. A different model of KSHV oncogenesis involving bone-marrow-derived mesenchymal stem cells demonstrated a dependence on inflammatory “KS-like” culture conditions to promote epigenetic de-repression and subsequent transformation [123]. Viral interleukin-6 (vIL6), a KSHV lytic gene, is a viral homolog of a host inflammatory cytokine that promotes tumorigenic growth [124]. Viral G protein-coupled receptor (vGPCR), another lytic gene, has been shown to contribute to inflammatory processes and promote proliferation and tumorigenicity of infected cells [125,126]. In addition to directly modifying tumorigenic potential, the KSHV lytic cycle may play more mechanical roles to promote the persistence and progression of KSHV-associated disease (Figure 4). In the context of KSHV-associated endothelial cells, lytic replication may be necessary to re-seed a reservoir of latently-infected cells and ensure consistent infection [127].

While the KSHV life cycle shapes and promotes the progression of KSHV-associated disease, it also represents an attractive therapeutic target (Figure 4). Cells undergoing complete lytic replication do eventually undergo cell death, and they are also sensitive to viral DNA synthesis inhibitors such as ganciclovir. Forcing the virus out of latency could represent a way to selectively target infected cells and address the clinical problem that persistent latent KSHV infection poses. Several groups have used animal models to demonstrate the potential of this approach. Cambogin, a bioactive natural product, has been shown to disrupt KSHV latency by preventing the interaction of LANA with SUMO, reducing the levels of viral episomes in infected cells. This disruption inhibited the proliferation of KSHV-infected cells in vitro and induced regression of PEL xenograft tumors in mice [128]. Inhibition of the MALT1 protease, a crucial facilitator of vFLIP- and K15-mediated NF-κB activation, triggered lytic activation and reduced growth and survival of PEL cells both in vitro and in a xenograft model [36]. Combination therapy composed of the protein kinase C activator PEP005 and the bromodomain and extra terminal (BET) protein inhibitor JQ1 robustly induced KSHV lytic reactivation, inhibiting PEL growth in in vitro and in vivo models [129]. Another combination therapy that has demonstrated efficacy in prolonging survival of PEL xenograft-bearing mice is bortezomib, a proteasome inhibitor, combined with vorinostat, an HDAC inhibitor. Bortezomib blocks late lytic gene expression which would normally be triggered by HDAC inhibition, leading to cell death without an increase in viremia [130]. Hence, disruption of latency represents a promising therapeutic target.

## 6. Conclusions

Due to millions of years of co-evolution with the molecular machinery of its host, KSHV has acquired manifold mechanisms to regulate its life cycle to optimize viral persistence and spread. These factors encompass epigenetic modifications, regulation by the host-cell-intrinsic and systemic immune defenses, and responses to environmental stimuli through regulation by host signaling pathways. Most importantly, this complex web of regulation creates a balance between the two life phases, which is critical to KSHV survival. Latency provides an opportunity for the virus to persist invisibly with minimal gene expression until certain stimuli allow the virus to massively upregulate lytic gene expression and progeny virion synthesis. KSHV maintains this balance in the context of malignant disease, persisting through low levels of lytic reactivation that sustain the latently-infected cell population and provide pro-growth paracrine factors. However, this delicate balance is ripe for therapeutic disruption. The complexity of regulation of the life cycle of KSHV offers both a challenge to our scientific understanding and a wide variety of potential options for intervention. However, as we gain a more detailed understanding of this regulation, we will become more capable of evaluating which angles of attack on the viral life cycle are most promising for the treatment of KSHV-associated disease.
